# Emotional Speech Recognition Method Based on Word Transcription

**DOI:** 10.3390/s22051937

**Published:** 2022-03-02

**Authors:** Gulmira Bekmanova, Banu Yergesh, Altynbek Sharipbay, Assel Mukanova

**Affiliations:** 1 Faculty of Information Technologies, L.N. Gumilyov Eurasian National University, Nur-Sultan 010008, Kazakhstan; gulmira-r@yandex.kz (G.B.); sharalt@mail.ru (A.S.); asiserikovna@gmail.com (A.M.); 2 Higher School of Information Technology and Engineering, Astana International University, Nur-Sultan 010000, Kazakhstan

**Keywords:** emotion recognition, speech recognition, crowd emotion recognition, affective computing, distance learning, e-learning, artificial intelligence

## Abstract

The emotional speech recognition method presented in this article was applied to recognize the emotions of students during online exams in distance learning due to COVID-19. The purpose of this method is to recognize emotions in spoken speech through the knowledge base of emotionally charged words, which are stored as a code book. The method analyzes human speech for the presence of emotions. To assess the quality of the method, an experiment was conducted for 420 audio recordings. The accuracy of the proposed method is 79.7% for the Kazakh language. The method can be used for different languages and consists of the following tasks: capturing a signal, detecting speech in it, recognizing speech words in a simplified transcription, determining word boundaries, comparing a simplified transcription with a code book, and constructing a hypothesis about the degree of speech emotionality. In case of the presence of emotions, there occurs complete recognition of words and definitions of emotions in speech. The advantage of this method is the possibility of its widespread use since it is not demanding on computational resources. The described method can be applied when there is a need to recognize positive and negative emotions in a crowd, in public transport, schools, universities, etc. The experiment carried out has shown the effectiveness of this method. The results obtained will make it possible in the future to develop devices that begin to record and recognize a speech signal, for example, in the case of detecting negative emotions in sounding speech and, if necessary, transmitting a message about potential threats or riots.

## 1. Introduction

Automatic recognition of human emotions is a new and interesting area of research. Achievements in the field of artificial intelligence are used in the development of affective computing and creation of emotional machines [1,2,3].

Today, research is being carried out on recognizing facial emotions from videos [4,5,6,7,8,9,10,11,12,13,14], on determining emotions by voice rh2ythm from audio information [15,16,17,18], and by writing style from texts [19,20,21]. In the following works [22,23], a deep convolutional neural network is used to recognize facial emotions from videos and images.

Today, the speech emotion recognition system (SER) assesses the emotional state of the speaker by examining his/her speech signal [24,25,26]. Work [27] proposes key technologies for recognition of speech emotions based on neural networks and recognition of facial emotions based on SVM, and in paper [28], they show a system of emotion recognition based on an artificial neural network (ANN) and its comparison with a system based on the scheme Hidden Markov Modeling (HMM). Both systems were built on the basis of probabilistic pattern recognition and acoustic phonetic modeling approaches. Work [29] presents a novel end-to-end convolutional neural network for age and gender recognition (CNN) with a specially designed multi-attention module (MAM) from speech signals to solve the problem of classifying native speakers according to their age and gender in speech processing. In the following works, when recognizing emotions, they use hidden Markov models for speech recognition [30,31,32,33].

According to the results of the analysis of studies, emotions play a vital role in e-learning [5,34,35]. It is also possible to determine users’ moods using affective computing methods [36] and use the result of moods to support the teacher by providing feedback relevant to learning.

For example, paper [37] describes a model that determines the mood of users using methods of affective computation and uses the results of mood to support the teacher by providing feedback related to teaching. Experienced teachers can change their teaching style according to the needs of the students.

With the help of the definition of emotion, it is possible to analyze the interests of the students and the learning outcomes of the course. Emotions can be detected by facial expressions [37,38]. Article [39] proposed a multimodal emotion recognition system that relies on speech and facial information. For the speech-based modality, they fine-tuned the CNN-14 of the PANNs framework, and for facial emotion recognizers, they proposed a framework that consists of a pre-trained Spatial Transformer Network on saliency maps and facial images followed by a bi-LSTM with an attention mechanism. Results on sentiment analysis and emotion recognition in the Kazakh language are published in [40,41,42,43,44].

The rest of the article is organized as follows: the introduction presents the most recent studies on the recognition of speech emotions. Section 2 describes the problem and proposes a solution for our methods. In Section 3 and Section 4, the experimental results and discussion are presented. Finally, in Section 5, we conclude our work.

## 2. Problem Description and Proposed Solution

With the development of modern speech technologies, a fundamental possibility has appeared as a transition from formal languages—intermediaries between man and machine to natural language in oral form as a universal means of expressing the goals and desires of a person.

Recognizing emotions in speech is a difficult problem. The opposite task to it is the synthesis of emotional speech. Moreover, the approaches for recognizing emotions in speech and the synthesis of emotional speech can be used the same. The solution to the problem of recognizing emotions in speech is difficult to separate from the problem of speech recognition itself, since the recognition of emotions is often associated with determining the meaning of what has been said. At the same time, there is currently no effective system for recognizing emotional Kazakh speech. Simplified, we can divide the solution of the problem into two parts, directly into speech recognition and emotion recognition. Previously, work was carried out on speech recognition based on a simplified transcription for the Russian language under the leadership of Shelepov V.Yu. in works [45,46,47,48,49], and for Kazakh speech in works [50,51]. The method was tested and published for the Russian and Kazakh languages and was also tested, but not published, by the authors for the English language, and it allows one to find words of a given structure after acoustic tuning for any language that has vowel (pronounced with the participation of vocal cords) and consonant (voiced and sonorous, pronounced with the participation of the vocal cords; deaf, pronounced without the participation of the vocal cords) sounds. This method allows one to find the given words in recognizable speech; in the case of emotion recognition, it makes it possible to find words that convey emotions. Therefore, this article discusses the issue of definitions of emotion from audio data. The method considered in this work is applied to the Kazakh language, but it can also be applied to any other language, only taking into account the definition of a list of words that carry emotions.

The Kazakh language (ISO 639-3, kaz) belongs to agglutinate languages and belongs to the Kypchak group of Turkic languages. The emotional coloring of the Kazakh speech is given by adjectives, verbs, and some types of nouns, as well as interjections (exclamations) expressing some emotions.

Shown below is a method for recognizing students’ emotional speech during online exams in distance learning—a model for determining emotions.

### 2.1. Emotional Speech Recognition Method

**Figure 1:**
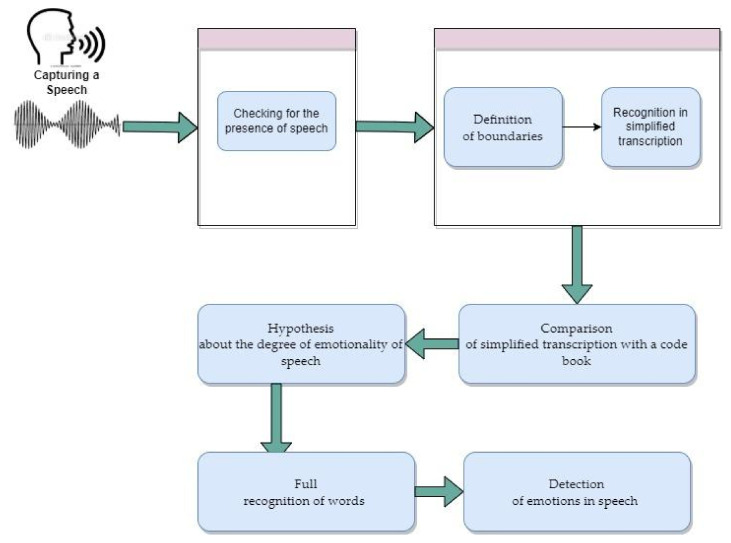
Emotional speech recognition process.

The emotional speech recognition method analyzes speech for the presence of emotions. The proposed method first assumes signal recognition, detection of speech in it, recognition in simplified transcription, definition of word boundaries, comparison of simplified transcription with a code book, and a hypothesis about the degree of emotionality of speech. In case of the presence of emotions, there occurs a full recognition of words and detection of emotions in speech (Figure 1).

The use of a codebook allows one to use this method with low computing power. The described method can be applied when there is a need to recognize positive and negative emotions in a crowd, in public transport, schools, universities, etc. This makes it possible to develop devices that begin to record and recognize a speech signal, for example, in the case of detecting negative emotions in sounding speech and, if necessary, transmit a message about a potential threat or a riot.

In this work, we tested the method on multi-user datasets to find solutions to the following research question:

Is it possible to effectively recognize emotions in speech based on a database of emotionally charged Kazakh words?

#### 2.1.1. Stages of Speech Signal Recognition

Before recognizing an emotion, there is a need to recognize the speech signal, i.e., convert spoken speech into text.

Recognition of a speech signal can consist of several stages: signal capture; definition of word boundaries; calculation of a sequence of quasi-periods; checking the recorded material for the presence of speech; clarification of the boundary of the speech end [46].

##### Capturing an Audio Signal

Capturing and digitizing an audio signal is carried out in accordance with the following parameters: 8 bit audio signal with a sampling rate of 22,050 Hz, such that its values have gradations from 0 to 255.

When a speech signal is input, 30,000 samples of “silence” are recorded, and successive segments of 300 samples each are analyzed in the recorded signal. For each of them, the following ratio is calculated [52]

(1) V/C

where

(2) $$V=\overset{298}{\underset{i=0}{\sum }}\left|x_{i+1}-x_{i}\right|$$

This is a numerical analogue of full variation, C is the number of points of constancy, that is, points in time for which, at the next moment, the magnitude of the signal remains unchanged. The value of the variable (1), typical for the used sound card, is automatically determined as the most frequently encountered in the array of values. Further, this value, increased by 0.1, is used as a characteristic of the “silence” for a particular sound card and noise level.

##### Checking a Signal for Speech

The recorded audio signal needs to be processed such that its boundaries (beginning and end) can be determined.

**Figure 2:**
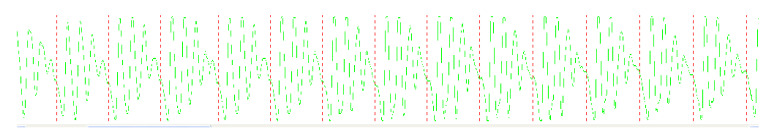
The result of automatic splitting of the voice section of the signal into quasi-periods.

The method for determining the beginning and end of speech is based on the fact that speech is quasiperiodic, since it is formed by trembling of the vocal cords [52]. Vowel and voiced consonants are formed using the vocal cords, which, under the action of the air flow from the lungs, vibrate quite periodically. Therefore, the corresponding signals (amplitude-time representations) are quasiperiodic. For example, this is how the amplitudetime representation of sound A in the word “ata” (“ata”—grandfather) appears (Figure 2):

This figure demonstrates vertical marks placed by a program that calculates successive quasi-periods according to the following algorithm.

The value is calculated.

(3) $$L_{k}:\;=\overset{k-1}{\underset{i=0}{\sum }}\left|x_{n_{0}+i}-x_{n_{0}+i+k}\right|$$

where $n_{0}$-the number of the sample from which the current quasiperiod began in the buffer,

(4) MIN≤k≤MAX

Here, we determine $k=k_{0}$, at which the value $L_{k}$ takes the minimum value. By definition, $k_{0}$ is the length of the quasiperiod. The *MIN* and *MAX* numbers are determined by the speaker’s pitch. *MIN* and *MAX* are determined depending on the speaker’s pitch; for example, for a tenor, they are respectively equal to 60 and 200.

Taking the end of the found quasiperiod as the beginning of the next one, we find the second quasiperiod, and so on. This makes it possible to distinguish quasi-periodic sections from non-quasi-periodic ones and to use this difference when checking the signal for the presence of speech.

Based on this method, the beginning and end of speech is determined.

Next, the speech recognition algorithm works up to simplified transcription.

To construct a simplified transcription, it is necessary to consider the operation of the transcriptor.

#### 2.1.2. Development of an Automatic Transcriptor

An automatic transcriptor is a program for translating any spelling text into a transcriptional record and vice versa based on linguistic rules.

A transcriptor of words to a transcriptional notation is present in all natural language processing systems such as translators, speech recognition or speech synthesis systems. However, depending on the intended purpose of the system, one or another transcriptor model is selected. Thus, for example, for speech recognition systems, a simplified transcription can be used, while for speech synthesis systems, it is necessary to use the most detailed transcription containing the acoustic characteristics of sounds.

**Figure 3:**
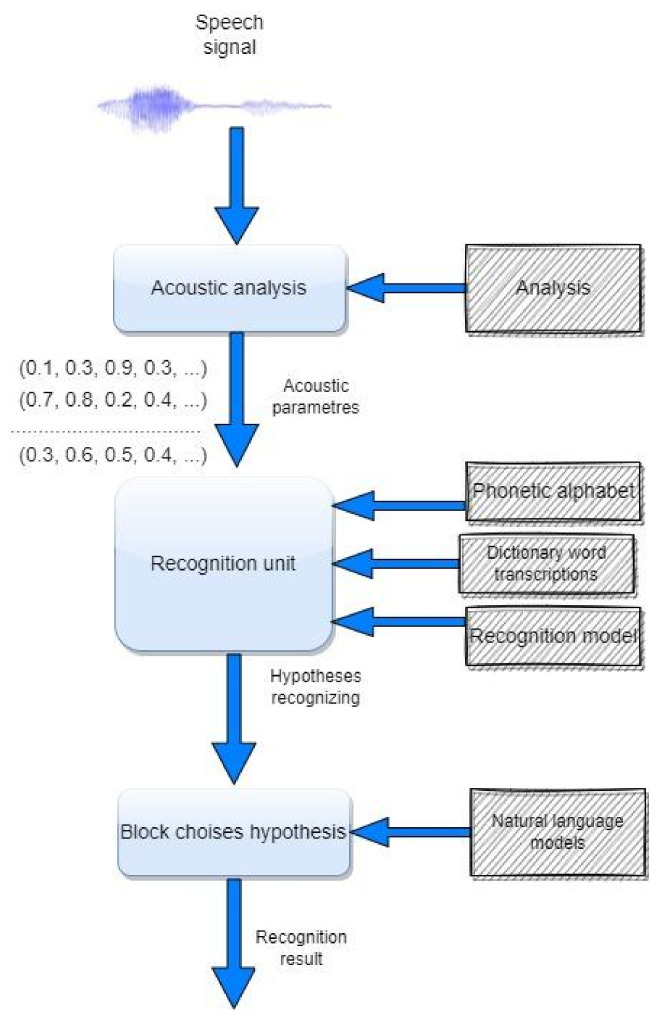
Kazakh speech recognition system.

This transcriptor model was developed for automatic Kazakh speech recognition systems (Figure 3).

The phonetic alphabet is the basis of the speech recognition unit. The symbols of the phonetic alphabet must unambiguously correspond to those sounds, the distinction of which is essential in the process of recognition. Therefore, the minimization of the size of the phonetic alphabet without compromising the quality of recognition should be carried out by identifying in the alphabet only those sounds that are the closest in sounding from the person’s point of view [45].

Therefore, for the correct construction of the transcriptor and the use of the phonetic alphabet, we first need to translate the record of the word into an intermediate alphabet of 31 letters, which are characteristic of the Kazakh language. At the second stage, the word recording is translated according to the formalized linguistic (phonological) rules of the Kazakh language [50]. At the third stage, the received words are translated into transcriptional notation in symbols of international transcription.

The current Cyrillized alphabet of the Kazakh language contains 42 letters instead of 31 letters [53]. Of these, 11 letters were mistakenly introduced in 1940 only such that the writing and reading of Russian words in the Kazakh text was carried out in accordance with the norms of the Russian language. These include: Ё, И, Ч, Щ, Ц, Һ, Э, Ю, Я, Ь, Ъ.

Therefore, for the correct construction of the transcriptor and the use of the phonetic alphabet, we first need to translate the record of the word into an intermediate alphabet of 31 letters. Correspondence of symbols with transcription is given in Table 1.

#### 2.1.3. Formalization of Phonological Rules of Sound Combinations in the Kazakh Language

There are nine vowel and 22 consonant sounds in the Kazakh language.

The formalization of phonological rules is described in detail in [50]; in general, in the Kazakh language, there is a law of syngharmonicity, when soft affixes are attached to the soft stem of a word (with soft vowels), and hard affixes are attached to the hard stem of the word. In addition, in the Kazakh language, there are rules for voicing and stunning consonants; for example, the word “aq manday” sounds similar to “agmanday”. There are seven similar rules for the Kazakh language.

A simplified transcription is automatically built based on the complete transcription of a word.

#### 2.1.4. Structural Classification of Kazakh Words and Use of Generalized Transcriptions

For good representation, in the structural classification, the symbols of the language are divided into classes, presented below. (Table 2).

As expected, there are few words with the same structure, for example, for a dictionary of 41,791 words, there are only three words with the CWCCWCW structure (Table 3).

Next, the signal is segmented to create a generalized transcription as described in [46,47,48,54].

This algorithm could help to develop even more generalized transcription, namely, to divide all the sounds into two natural classes: vowels and consonants. Such a division gives good results in little vocabularies as well.

##### Construction of Averaged Standards

In order to reduce the dependence of the recognition system on the speaker, we applied the procedure of averaging the standards spoken by several speakers. For example,

(5) $$E=\left(e_{1},e_{2},\ldots ,e_{27}\right)$$

(6) $$A=\left(a_{1},a_{2},\ldots ,a_{27}\right)$$

two standards of the same word, and for the sake of generality, we will assume that the standard has already been obtained by averaging the standards spoken by *n* speakers, and *a* is the standard of the *n* + 1st speaker. We take a vector and then $a_{j}+\ldots +a_{j+k}$, which is all the corresponding vectors from set (5) in the meaning described above. Then we place

(7) $$e_{i}^{’}=\frac{n}{n+1}e_{i}+\frac{n}{n+1}\frac{a_{j}+\ldots +a_{j+k}}{k}$$

Calculating this for all *i* = 1, 2, … 27, we obtain the result of averaging the standards for *n* + 1 speakers:

(8) $$E^{\prime }=\;\left(e_{1}^{’},\;e_{2}^{’},\ldots ,\;e_{27}^{’}\right)$$

The coefficients

(9) $$\frac{n}{n+1},\;\frac{1}{n+1}$$

were introduced in order to make all speakers equal. However, as the number n increases, the changes introduced by new speakers become smaller and smaller. The same procedure allows for averaging several standards of one speaker in order to increase their reliability. The effectiveness of this procedure becomes especially clear if we apply it to averaging the standards of different words. Thus, for example, it allows one to teach the computer to perceive each word of the line

“Ақ киімді, денелі, ақ сақалды,”

as symbol 0, and each word of the line

“Сoқыр мылқау танымас тірі жанды”

as symbol 1, constructing for them the corresponding averaged standards.

#### 2.1.5. Codebook and Its Construction Technique

When solving the problem of determining emotions in speech, it is proposed to use the technique of constructing a code book, which allows for significantly narrowing the search for candidates for recognizing emotionally charged words. The knowledge base of emotionally charged words is presented in the form of a code book.

Storing the patterns described above containing arbitrary vectors requires a lot of memory. It can be significantly reduced, and at the same time, a significant gain in recognition speed is obtained by using a relatively small set of so-called code vectors instead of arbitrary vectors. These latter are used to approximate arbitrary vectors and construct a code book. Code vectors are also referred to as codebook words [46].

To construct a codebook with *M* size, the so-called *k*-means method is used.

1. Initialization:

From the number of *L* training vectors, we arbitrarily choose *M* vectors as the initial set of words in the code book.

2. Finding the nearest neighbor:

For each training vector, we find the closest codebook vector. The set of training vectors “gravitating” in this sense to the same code vector will be called its corresponding cell.

3. Modernization with a centroid:

For each cell, we replace the corresponding code vector with the centroid (average) of the set of training vectors that fall into this cell.

4. Iteration:

We repeat steps 2 and 3 until the sum of the distances of all training vectors to the corresponding code words stops decreasing by more than a predetermined threshold.

Although the described method of constructing a codebook works quite well, it has been shown that it is more expedient to build a codebook by increasing its dimension step by step. Starting with a book with one code vector and successively doubling the number of code vectors using the splitting method. This procedure is called a binary splitting algorithm and can be described as follows:

1. We create a codebook from one word, taking for it the centroid of the set of all training vectors.

2. Then, double the size of the codebook by splitting each code vector according to the rule.


(10) $$y_{1}=\;\left(1+\varepsilon \right)y$$

(11) $$y_{2}=\;\left(1-\varepsilon \right)y$$

here, ε is the splitting parameter with a value from 0.01 to 0.05.

3. Use the k-means Algorithm to obtain the best possible set of code vectors for a double-size codebook.

4. Repeat steps 2 and 3 until we obtain the codebook of the required size.

The dimension of a codebook constructed in this way is a power of 2.

#### 2.1.6. Recognizer Using a Codebook

The method for constructing standards using a codebook consists of replacing each of the 27 vectors included in the standard with the nearest code vector (in the meaning of the *l*_1_ metric described above). Then, it becomes possible to store the standard in the form of a sequence of numbers of the corresponding code vectors. This, taking into account the need to store the codebook, gives a significant memory savings with a sufficiently large vocabulary. Further, the recognition process is built as follows. The word to be recognized is written as a set of 27 arbitrary (non-code) vectors. Then, a table of the distances of these vectors to all the vectors of the codebook is built. Next, the DTW-distances of the considered word to all standards are calculated. In this case, the distances between the vectors are taken from the mentioned table and are not calculated every time as it was when the codebook was not used. This consumes significantly less time. Thus, a significant gain is achieved, both in recognition speed and in the amount of required memory.

#### 2.1.7. Step Recognition Algorithm

If a large vocabulary is recognized and the number of standards is large, then recognition by full comparison of what has been said with each of them is too long of a process. Accelerating it and at the same time increasing the recognition reliability can be possible by our proposed “Stepwise Recognition Algorithm”. Let us describe it using a vocabulary of 12,000 emotional words. Its essence is as follows. First, the spoken word is compared with all DTW-based standards, but only the first two thousand samples are involved in the recognition. The result is a list of 180 closest candidate words (this number may vary depending on the size of the original vocabulary. For the mentioned vocabulary, it seems to be optimal). Further, recognition is carried out within this list using the first four thousand samples, as a result of which the list of candidates is halved. Then the same is performed sequentially for segments of six thousand, eight thousand and, finally, ten thousand samples. We arrived at this algorithm, which provides faster and more reliable recognition of large vocabularies, as follows. At first, a system was made that worked with voice input with preliminary selection of a sufficiently short segment of the recognized vocabulary by typing one, two or three initial characters of the word being entered on the keyboard. Convinced of the high reliability of this method, we noticed that close words (words with a similar beginning) are recognized. Recognition of words with different origins should be even more reliable, and recognition on a shortened initial segment is sufficient to isolate this beginning [49,50].

### 2.2. Defining Emotions

The classification uses the six main classes of emotions that correspond to Ekman’s classification, such as anger, fear, disgust, happiness, sadness and one neutral class. The neutral class includes all cases that could not be defined for other classes.

Table 4 shows a fragment of the emotional vocabulary of the Kazakh language:

The works in [41] describe methods for determining the polarity of the text. When analyzing sentiment and emotion, the results of morphological [55,56] and syntactic analysis of the Kazakh language [57,58,59] are used.

To apply this method to other languages, it is necessary to determine the list of words that give emotional coloring to speech, and then the proposed method will, after performing simplified speech recognition, find words that have the same structure as words with emotional coloring and a hypothesis will be put forward that the sounding speech is emotional and there is a need to perform full-fledged speech recognition, which is demanding on computing resources.

#### 2.2.1. Construction of Emotion Vocabulary Generalized Transcriptions

The emotion vocabulary allows for constructing its generalized transcriptions and applying them to search for words from emotion vocabulary. Examples of emotional Kazakh words with generalized transcription are shown in Table 5.

An example of a database of emotional words of the Kazakh language and speech recognition demo code can be downloaded here.

The simplified transcription in this article has two main purposes. First, simplified transcription is used in the speech recognition task. A simplified transcription is built, as for example, the word *ata* (ata—grandfather) is similar to CPC (vowel + voiceless consonant + vowel). In the task of speech recognition, this simplified transcription is used to search for possible candidates for recognition from the dictionary of all words of the Kazakh language that have simplified transcriptions (in our work, these are more than three million, two hundred thousand word forms of the Kazakh language). For our example, the word *apa* (apa—grandmother) has the same simplified transcription CPC (vowel + voiceless consonant + vowel). A list of candidates for recognition is compiled, and then a recognition algorithm based on the code book is run. Second, simplified transcription is used to determine the presence of emotionally colored words in speech and can significantly reduce the search time. At the initial stage, the algorithm allows for understanding of whether a speech, audio recording or text contains emotionally charged words. Thus, for example, the word “уай”—(way) is the equivalent of “wow” in English; it has a simplified transcription of WWC, which occurs only once in the entire dictionary of emotionally charged words.

#### 2.2.2. Emotion Recognition Model

A production model is proposed for modeling emotion recognition from Kazakh texts. To build a production model, the following meta-designations are introduced (Table 6):

Below is a model based on formalized production rules for determining the emotion of lexical units (phrases) in a Kazakh text. Below are examples of the rules for defining emotions in the Kazakh language.

1. If a lexical unit contains a noun with the emotional color of happiness and the next word after it is a verb (of a positive form) with a neutral tone, then the emotional description of this phrase is happiness.

(12) $$\frac{\omega \in L,\;\omega =\zeta \cdot \alpha \cdot \beta \cdot \xi ,\;\alpha \in N,\;emo\left(a\right)=happiness,\;\beta \in V_{Post},\;emo\left(\beta \right)=0}{\;emo\left(\omega \right)=happiness\;}$$

Here and below ζ, ξ—any strings of words, including empty ones. For example, шабыт келді (ʃɑbɯt kеldɪ).

2. If a lexical unit contains an adjective describing the emotion anger and the next word after it is a verb (positive form) with a neutral tonality, then the emotional description of this phrase is anger.

(13) $$\frac{\omega \in L,\;\omega =\zeta \cdot \alpha \cdot \beta \cdot \xi ,\;\alpha \in Adj,\;emo\left(a\right)=anger,\;\beta \in V_{Post},sent\left(\beta \right)=0\;}{emo\left(\omega \right)=anger}$$

For example, тиянақсыз бoлды (tiyɑnɑqsɯz bɔldɯ).

3. If a lexical unit contains an interjection with an emotional connotation sadness, then the emotional description of this phrase is sadness.

(14) $$\frac{\omega \in L,\;\omega =\zeta \cdot \alpha \cdot \beta \cdot \xi ,\;\alpha \in Intj,\;emo\left(a\right)=sadness,\;\beta \in ,\;sent\left(\beta \right)=0}{emo\left(\omega \right)=sadness}$$

For example, масқара(mɑsqɑrɑ), қап(qɑp).

The use of formal rules for determining emotions allows for extracting emotions from speech recognized in the text based on a codebook.

## 3. Experiment

In our research, words that express emotions are highlighted and divided into six classes (Table 7).

The table shows the classes of emotions, the main ones of which correspond to Ekman, according to which students’ speeches were classified. This classification has been applied in previous works and has shown its effectiveness.

For the experiment, 420 audio recordings were studied, in which the greatest activity (voice acting) was found during the online exam.

At the moment for the Kazakh language, there are no sufficiently published results of works on emotional recognition or synthesis of Kazakh speech. Therefore, it is currently not possible to evaluate the proposed method in the traditional way. Thus, the evaluation was carried out as follows. Accuracy was evaluated for the Kazakh language using the Emotional Speech Recognition Method and DNN model presented in [60] (Table 8).

When recognizing emotions in the Kazakh language, an existing dictionary of 12,000 words was used. The emotionally colored dictionary passed an expert evaluation of the polarity values by the employees of the Research Institute “Artificial Intelligence”, taking into account the context of the task. The Research Institute employs linguists/philologists, experts in the field of artificial intelligence, as well as students of the Faculty of Information Technology who perform research practice at this Research Institute.

The model in [60] recognized the emotions with an accuracy of 82.07%. The model often confuses the emotions anger with happiness, which is related to extremely high emotional coloring. According to the published data, the result was 2.37% more, while the number of emotion classes was three classes less compared to our work.

## 4. Results

To recognize emotions, we used audio from video recordings of taking online exams during examination sessions. The database contains about 20,000 videos, which contain the speech of students and their comments during and after the exam. However, many audio recordings do not contain emotional speech, since during the online computer test, students restrain and do not express emotions.

We have studied 420 audio recordings. Audio recordings of exams were used, which were automatically processed by the speech recognition program and converted into text. All converted text was translated into a simplified transcription. Further, in the dictionary of emotionally colored words, a search for the spoken text was carried out using a simplified transcription. That is, we looked for emotionally charged words in audio recordings. If the text of the audio recording contained emotionally colored words, the text was further processed using the Emotion Recognition Model. That is, the emotions of the text were automatically placed by the program, and the audio recordings received tags with emotions, which were then manually checked by human taggers for the purity of the experiment. In 79.7% of cases, emotionally charged words were found correctly.

**Figure 4:**
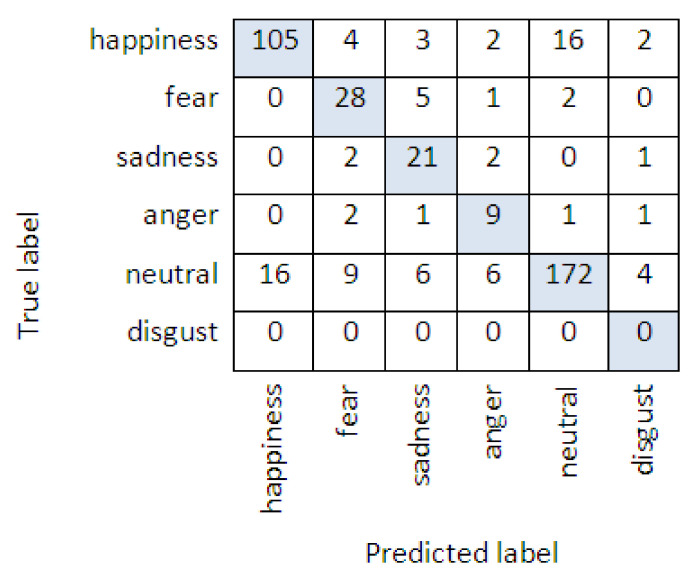
Confusion matrix of Emotional Speech Recognition Method.

The confusion matrix of Emotional Speech Recognition Method for the Kazakh language is presented in Figure 4.

With the proposed Emotional Speech Recognition Method, we reached an accuracy of 79.7%. According to the results of the experiment, 105 students who received the expected good grade expressed happiness; there were students who were afraid during the exam or worried, and 28 were classified as fear. Further, after the output of the exam results, students clearly expressed sadness and anger if they did not obtain a good mark on the exam. During the experiment, the proposed recognition method confused the classes of fear, sadness and anger emotions. This was due to the semantic proximity of the used emotionally colored words. Moreover, 172 students were assigned as neutral, since they did not voice their reactions. In view of the fact that during the computer test, students did not speak much and did not express emotions much, the emotion classes proposed in Table 4 were considered sufficient by the authors of the article.

## 5. Discussion

The results of this work were from a method for recognizing emotional speech based on the emotion recognition model and the emotional vocabulary of emotionally charged words of the Kazakh language with generalized transcription, as well as from a code book.

There are various works on recognizing emotions in speech, but they do not rely on the proposed model for determining emotions, and this work also showed for the first time the construction and use of an emotional vocabulary of emotionally charged words of the Kazakh language with generalized transcription, as well as a code book, which significantly reduced the time search and was not resource consuming, which expanded the scope of the proposed method. This method can be adapted for other languages as well if an emotional vocabulary is available.

The main disadvantage of methods for direct recognition of emotions in speech can be considered as the need to process speech before its recognition. Thus, most often, speech in preprocessing before recognition undergoes a smoothing procedure to eliminate random inclusions, noise, and also reduces the signal amplitude. The value of the amplitude of a speech signal, in addition to the loudness of speech, also reflects intonation. Such normalization of speech leads to the fact that the intonation of speech is smoothed out, which is also a means of expressing emotions. Consequently, an increase in the accuracy of recognizing what was said almost always leads to a decrease in the accuracy of recognizing emotions in spoken speech. Thus, methods that allow speech recognition to be carried out with high accuracy must be able to recognize emotions in some way.

In the future, it is planned to explore the possibilities of recognizing emotions from images and videos accompanying the recognized audio signal. In addition, the obtained results will allow one in the future to solve the problem of the synthesis of emotional speech on the basis of the emotional vocabulary of emotionally charged words.

## 6. Conclusions

Speech recognition technologies and emotion detection contribute to the development of intelligent systems for human–machine interaction. The results of this work can be used when it is necessary to recognize positive and negative emotions in a crowd, in public transport, schools, universities, etc. The experiment carried out has shown the effectiveness of this method. The results obtained will allow one in the future to develop devices that begin to record and recognize a speech signal, for example, in the case of detecting negative emotions in speech and, if necessary, transmitting a message about a potential threat or a riot.

In this paper, we presented a method for recognizing emotional speech based on an emotion recognition model using the technique of constructing a code book. The code book allows for significantly narrowing the search for candidates for recognizing emotionally charged words. Answering the research question, we can say that it is possible to effectively recognize the emotions of the Kazakh language based on a database of emotionally charged words. An emotional vocabulary of the Kazakh language was built in the form of a code book. The use of this method has shown its effectiveness in recognizing the emotional speech of students during an exam in a distance format with an accuracy of 79.7%. At the same time, there are certain risks in terms of speech recognition, which must be effective. Its effectiveness directly depends on the level of noise and the use of grammatically correct language (no slang, no use of foreign words in speech, etc.). There are also risks associated with incorrect recognition of emotions, which may result from the incompleteness of the emotional vocabulary. In addition, the meanings of polarity in emotional vocabulary may need to be automatically reconfigured in the context of subject areas. For example, polarity meanings should differ when recognizing the emotions of a crowd at a rally from the polarity emotions of a student taking an exam, etc. This raises questions about verifying the correctness of automatically configured polarity. This gives food for thought and the formulation of new research topics.

In the future, we can think about using other types of features and apply our system on other bases, including larger ones, and using a different method to reduce the size of the feature. Finally, we can also consider emotion recognition using an audiovisual base, and in this case, benefit from descriptors from speech and others from image. This allows for improving the rate of recognition of each emotion.

## Figures and Tables

**Table 1:** Symbols of the current alphabet and intermediate alphabet.

Current Alphabet	Intermediate Alphabet	Transcription	Current Alphabet	Intermediate Alphabet	Transcription
А	А	(ɑ)	Б	Б	(b)
Ә	Ә	(æ)	В	В	(v)
Е	Е	(е)	Г	Г	(g)
О	О	(ɔ)	Ғ	Ғ	(ɣ)
Ө	Ө	(ɵ)	Д	Д	(d)
Ұ	Ұ	(ʊ, u)	Ж	Ж	(Ʒ)
Ү	Ү	(ү)	З	З	(z)
Ы	Ы	(ɯ)	Й	Й	(y)
І	І	(ɪ, i)	К	К	(k)
Э	Е	(jɪ)	Қ	Қ	(q)
Я	ЙА	(yɑ)	Л	Л	(l)
Ю	ЙУ	(yw)	М	М	(m)
Ё	ЙО	(yɔ)	Н	Н	(n)
И	ІЙ	(iy)	Ң	Ң	(ŋ)
И	ЫЙ	(ɯj)	П	П	(p)
Ч	Ш	(tʃ)	Р	Р	(r)
Щ	Ш	(ʃ)	С	С	(s)
Ц	С	(tc)	Т	Т	(t)
Һ	Х	(h)	У	У	(w)
Ъ	-		Ш	Ш	(ʃ)
Ь	-		Ф	Ф	(f)
			Х	Х	(h)

**Table 2:** Symbols of the current alphabet and intermediate alphabet.

Classes	Symbols	Meaning
W	аұыoеәүіөу	vowels and consonant «У»
C	бвгғджзйлмнңр	voiced consonants
F	сш	voiceless hush consonants
P	кқптфх	voiceless consonants

**Table 3:** Example of the words with a structure CWCCWCW.

Kazakh Word	Transcription	Generalized Transcription
бұлдану	bʊldɑnw	CWCCWCW
бұлдыра	bʊldɯrɑ	CWCCWCW
бүлдіру	bүldɪrw	CWCCWCW

**Table 4:** Fragment of the emotional vocabulary.

Word	Transcription	Translation	POS	Emotion
діріл	dɪrɪl	trembling	N	fear
қoрқақтық	qɔrqɑqtɯq	cowardice	N	fear
ақылсыз	ɑqɯlswz	stupid	N	anger
қызғаныш	qɯzɣɑnɯʃ	jealousy	N	anger
құрмет	qʊrmеt	honor	N	happiness
нәзіктік	næzɪktɪk	tenderness	N	happiness
шапшаң	ʃɑpʃɑŋ	quick	Adv	happiness
шарасыздан	ʃɑrɑsɯzdɑn	involuntarily	Adv	sadness
сөзқұмар	sɵzqʊmɑr	garrulous, chatty	Adj	disgust

**Table 5:** Example of words from emotion vocabulary with generalized transcription.

Word	Transcription	Translation	POS	Emotion	Generalized Transcriptions
қoрқақтық	qɔrqɑqtɯq	cowardice	N	fear	PWCPWPPWP
ақылсыз	ɑqɯlswz	stupid	N	anger	WPWCFWC
көз жасы	kɵz Ʒɑsɯ	tear	N	sadness	PWC CWFW
құрмет	qʊrmеt	honor	N	happiness	PWCCWP
шапшаң	ʃɑpʃɑŋ	quick	Adv	happiness	FWPFWC
шарасыздан	ʃɑrɑsɯzdɑn	involuntarily	Adv	sadness	FWCWFWCCWC
сөзқұмар	sɵzqʊmɑr	garrulous, chatty	Adj	disgust	FWCPWCWC
тату	tɑtw	amicably	Adj	happiness	PWPW
тиянақсыз	tiyyɑnɑqsɯz	fragile	Adj	anger	PWCCWCWPFWC
пішту!	pɪʃtw!	my gosh	Intj	disgust	PWFPW!
туу	tww	Holy	Intj	sadness	PWW
уай	wɑy	Wow	Intj	happiness	WWC
бұзықтық істеу	bʊzɯqtɯq ɪstеw	roughhouse	V	anger	CWCWPPWP WFPWW
бәрекелді	bærеkеldɪ	Bravo	Intj	happiness	CWCWPWCCW
әй	æy	hey	Intj	anger	WC
әттеген-ай	ættеgеn-ɑy	What a pity	Intj	sadness	WPPWCWC-WC
қап	qɑp	it’s a shame	Intj	sadness	PWP
масқарай	mɑsqɑrɑy	What a mess	Intj	sadness	CWFPWCWC
мәссаған	mæssɑɣɑn	Gee	Intj	fear	CWFFWCWC

**Table 6:** Meta-designations.

Designation	Purpose
α,β,γ,…,ζ,ξ, …,	Many words in the language—Variables
ω	ω=ζ·α·β·ξ—Lexical units (non-empty word or phrase)
L	Set of sentences in the language
N	Set of nouns
Adj	Set of adjectives
Pron	Set of pronouns
V_Post	Set of positive verb forms
V_Negt	Set of negative verb forms
Intj	Set of interjections
AdvIntens	Set of adverbs or enhancing
emo	Emotion Establishment—Predicate
@	Negation words “емес/жoқ”(no)—Constants
¬	Transformation to negative form—Operation
⋅	Concatenation—Operation

**Table 7:** Emotion classes.

Emotion Classes	Polarity	Example
happiness	positive	Алақай! Мен сәтті аяқтадым (Hooray! I finished successfully)
fear	negative	Жауаптарды ұмытып қалдым (I forgot the answers)
disgust	negative	Туу, oйдағыдай баға алмадым (Tuu, didn’t get the expected grade)
sadness	negative	Қап! кейбір жауапты білмей қалдым. (Qap, I didn’t know the answer.)
anger	negative	Кедергі жасама! Уақыт тығыз (do not bother, time is running out)
neutral	neutral	Бүгін барлығы емтихан тапсырады (everyone is taking exams today)

**Table 8:** Quantitative evaluation of the different methods on the speech emotion recognizer.

Method	Dataset Language	Number of Classes	Accuracy
Emotional Speech Recognition Method	Kazakh	6	79.7%
DNN model [59]	Kazakh, Russian	3	82.07%
